# Retraction Note: Ornithine-A urea cycle metabolite enhances autophagy and controls Mycobacterium tuberculosis infection

**DOI:** 10.1038/s41467-022-33608-y

**Published:** 2022-10-18

**Authors:** Ramya Sivangala Thandi, Rajesh Kumar Radhakrishnan, Deepak Tripathi, Padmaja Paidipally, Abul K. Azad, Larry S. Schlesinger, Buka Samten, Sachin Mulik, Ramakrishna Vankayalapati

**Affiliations:** 1 Department of Pulmonary Immunology, Center for Biomedical Research, University of Texas Health Center, Tyler, TX 75708 USA; 2 Texas Biomedical Research Institute, San Antonio, TX 78227 USA

**Keywords:** Autophagy, Tuberculosis, Monocytes and macrophages, Metabolism

**Retraction to**: *Nature Communications* 10.1038/s41467-020-17310-5, published online 15 July 2020

The Editor has retracted this Article at the authors’ request. Following concerns from a reader, the authors initiated an internal investigation. After comparing the laboratory record and the figures, it was concluded that:

- The﻿ data in Fig. 3c did not originate from three biological replicates as claimed in the legend;
- In Figs. 3c and 5f, multiple lanes could not be matched back to the original raw data in the laboratory records;
- The GAPDH bands in Figs. 3c, 5f and 6e could not be matched with raw data;
- The LC-3B image in Fig. 5f was duplicated and rotated 180 degrees in Fig. 6e to represent p-AMPK (T172);
- In Fig. 7d, the 4X and 10X images did not originate from the same samples;
- In Fig. 7d, the 10X images of Mtb H37Rv + Ornithine and Mtb H37Rv + Imidazole contain overlap.

Further data similarities have been identified in Figs. 2j and S1, as well as within Figs. S5, S10, S11 and S16. In light of these issues, the authors have requested to retract this Article.

Ramya Sivangala Thandi has not responded to any correspondence from the editor or publisher about this retraction. All other authors agree to this retraction.

